# Xanthogranulomatous pyelonephritis in a 47-day-old male infant: a case report

**DOI:** 10.3389/fped.2025.1625781

**Published:** 2025-07-10

**Authors:** Yeping Jiang, Menglin Chang, Qian Fu, Hui Wang

**Affiliations:** ^1^ Department of Nephrology, Beijing Children’s Hospital, Capital Medical University, Beijing, China; ^2^ Department of Nephrology, Baoding Hospital of Beijing Children’s Hospital, Capital Medical University, Regional Center for Children’s Health, Baoding, China

**Keywords:** xanthogranulomatous pyelonephritis, infant, metagenomic sequencing, escherichia coli, antibiotics

## Abstract

**Background:**

Xanthogranulomatous pyelonephritis (XGP), a rare granulomatous renal disease linked to bacterial infection (e.g., *Escherichia coli*), presents challenges in pediatric diagnosis, especially in infants, due to overlap with neoplastic renal masses like Wilms tumor.

**Case Summary:**

A 47-day-old male infant with fever, elevated inflammatory markers (WBC 13.94 × 10^9^/L, CRP 110.43 mg/L), and urinary leukocytes/hematuria showed a left renal mass (1.7 × 1.8 × 2.1 cm) on imaging. Biopsy revealed histiocytic-neutrophilic infiltration with focal necrosis, and metagenomic sequencing identified dominant E. coli. Antibiotic therapy (cefoperazone-sulbactam followed by cefdinir) induced regression (1.1 × 0.8 × 1.1 cm at 2 weeks). Elevated AFP (888.27 ng/ml) normalized, excluding malignancy.

**Conclusion:**

This case highlights XGP as a critical differential diagnosis for febrile infants with renal masses. Integration of histopathology, metagenomic sequencing, and prolonged follow-up confirms that focal XGP can be managed successfully with targeted antibiotics, avoiding nephrectomy.

## Introduction

Xanthogranulomatous pyelonephritis (XGP) is a rare, chronic granulomatous renal disease characterized by extensive inflammatory infiltration, typically associated with bacterial infections and often linked to urinary tract obstruction or stasis. While XGP is infrequently reported in pediatric populations, its presentation in infants poses significant diagnostic challenges due to clinical and radiological overlap with neoplastic entities such as Wilms tumor, congenital mesoblastic nephroma, or neuroblastoma. These malignancies must be rigorously excluded in infants presenting with renal masses, particularly when accompanied by systemic symptoms like fever or elevated inflammatory markers, which are nonspecific and may mimic both infectious and neoplastic processes. In pediatric patients, especially neonates and young infants, the clinical manifestations of XGP are often atypical, lacking the classic adult features such as flank pain or palpable masses. Instead, infants may exhibit nonspecific signs, including fever, poor feeding, or failure to thrive, necessitating a comprehensive diagnostic approach that integrates imaging, histopathology, and microbiological evaluation. We present a case of a 47-day-old male infant with XGP caused by E. coli, highlighting the importance of multimodal diagnostics in distinguishing this rare infectious entity from renal malignancies in infants. This case emphasizes the critical role of a high index of suspicion, combined with advanced diagnostic modalities, in the evaluation of febrile infants with renal masses to ensure timely and appropriate intervention.

## Case description

A 47-day-old male infant was admitted to the hospital with a 1-day history of fever. The infant had no prior infections, including urinary tract infections (UTI) or other systemic infections, confirmed by medical history review. During the illness, there was no hematuria, edema, night sweats, or weight loss. The preliminary diagnosis at admission was urinary tract infection. Laboratory studies revealed a white-cell count of 13.94 × 10^9^/L (neutrophils 51.8%), C-reactive protein (CRP) 110.43 mg/L, and procalcitonin (PCT) 1.97 ng/ml. Urinalysis showed leukocytes 3+, blood 3+, and microscopic examination of centrifuged urine sediment revealed 150–160 white blood cells per high-powered field. Alpha-fetoprotein (AFP) was elevated at 888.27 ng/ml. Routine immunological tests (serum immunoglobulin levels and T/B cell subsets) were performed during hospitalization, all showing normal results. Classic urine culture with antibiogram showed no bacterial growth.

**Figure 1:**
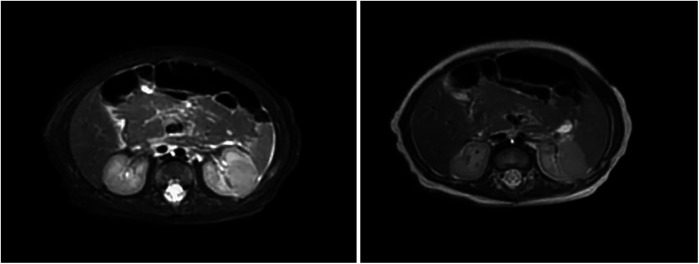
MRI images of the kidney. A well-circumscribed space-occupying lesion in the left kidney [isointense on T1-weighted imaging (T1WI), isointense on T2-weighted imaging (T2WI), slightly hyperintense on diffusion-weighted imaging (DWI), measuring approximately 1.7 × 1.8 × 2.1 cm] demonstrates heterogeneous enhancement. The renal calyces course along its superior margin, with no significant perilesional edema observed.

**Figure 2:**
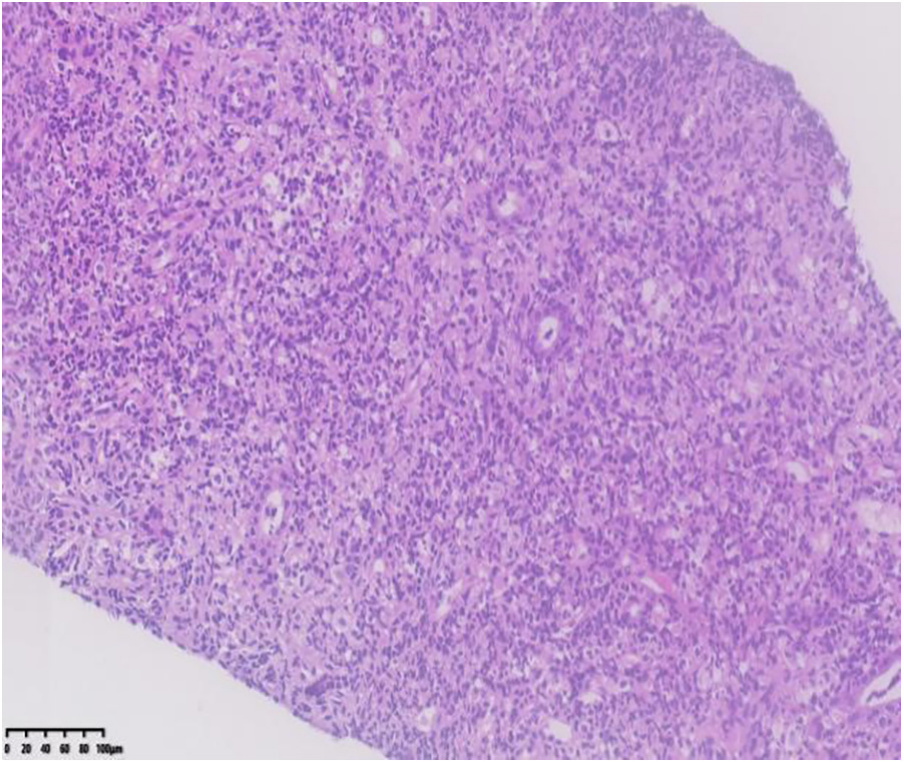
Kidney- biopsy specimen: histopathology demonstrated extensive interstitial infiltration by histiocytes, neutrophils, lymphocytes, and scattered eosinophils, with tubular epithelial involvement and focal necrosis.

**Figure 3:**
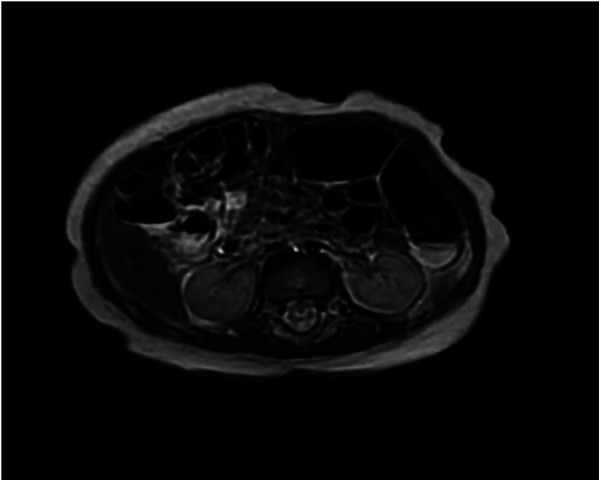
MRI images of the kidney (seven weeks post-discharge). T2-weighted fat-suppressed sequences within the left kidney. Both kidneys maintained normal dimensions, with the right kidney exhibiting no abnormal signal intensities.

Renal ultrasound: A space-occupying lesion in the mid-pole of the left kidney (total dimensions 1.6 × 1.0 × 1.2 cm) with a hyperechoic rim (thickness 0.3 cm) was observed, suggestive of infection. A central hypoechoic area raised suspicion for abscess formation, though neoplastic etiology could not be fully excluded. Pelvic and lower abdominal MRI: A rounded mass in the left kidney (isointense on T1 and T2-weighted imaging, slightly hyperintense on DWI, size 1.7 × 1.8 × 2.1 cm) was noted (Figure 1). Biopsy and pathology: Under ultrasound guidance, three core needle biopsies (18-gauge) were obtained from the left renal lesion. Grossly, the specimens were gray-white and measured 0.7–0.9 cm in length. Histopathology demonstrated extensive interstitial infiltration by histiocytes, neutrophils, lymphocytes, and scattered eosinophils, with tubular epithelial involvement and focal necrosis. Xanthogranulomatous pyelonephritis was suspected, though limited sampling necessitated clinical correlation and further microbiologic evaluation (Figure 2). Immunohistochemistry showed positivity for CD68, CD163, and WT1, with focal S-100 and CD138 staining. Ki-67 proliferation index was approximately 30%. Special stains (Gram, AFB, PAS) and EBER *in situ* hybridization were negative. Metagenomic sequencing of renal tissue identified Escherichia coli (Gram-negative bacilli, 1,044 sequences, 85.78% relative abundance). No viral, fungal, parasitic, or atypical pathogens were detected. Sequencing targeted ESBL-encoding genes (TEM, SHV, CTX-M), but no ESBL genes were detected. Intravenous cefoperazone-sulbactam was administered for 13 days, followed by oral cefdinir granules at therapeutic doses. Repeat renal ultrasound at 2 weeks post-discharge demonstrated reduction in the left renal lesion (1.1 × 0.8 × 1.1 cm) with a thinner hyperechoic rim (0.25 cm) and resolution of the central hypoechoic area. Seven weeks post-discharge (following two months of anti-infective therapy), magnetic resonance imaging (MRI) demonstrated patchy areas of slightly decreased signal intensity on T2-weighted fat-suppressed sequences within the left kidney. Both kidneys maintained normal dimensions, with the right kidney exhibiting no abnormal signal intensities (Figure 3). The extent of abnormal signal intensity in the left kidney was notably reduced, evidencing significant therapeutic improvement.

## Discussion

The presentation of a febrile infant with a renal mass and elevated inflammatory markers necessitates a broad differential diagnosis. Key considerations include acute pyelonephritis, which typically manifests with fever, leukocytosis, and pyuria but lacks the focal mass-like lesions seen here. Renal abscess, often secondary to untreated pyelonephritis, shares overlapping imaging features but may lack the granulomatous histopathology characteristic of xanthogranulomatous pyelonephritis (XGP). Wilms tumor and congenital mesoblastic nephroma, though rare in neonates, must be excluded due to their solid mass appearance; however, these tumors typically lack the inflammatory infiltrates and lipid-laden macrophages seen in XGP. Neuroblastoma may present with a retroperitoneal mass but is distinguished by elevated catecholamine metabolites and distinct histopathology. Renal tuberculosis, while unlikely given negative AFB stains and EBER testing, should remain a consideration in endemic regions. Less common entities include renal lymphoma (unlikely given age and CD68/CD163 positivity) and metanephric stromal tumor, which may exhibit stromal fibrosis but lacks the neutrophilic infiltrates and foam cells of XGP. The combination of E. coli-dominant metagenomic sequencing, histiocytic infiltration, and imaging evolution under antibiotics strongly favors XGP over neoplastic etiologies.

XGP is a rare renal disease characterized by chronic granulomatous inflammation. XGP results from a “failed” immune response to chronic infection, where macrophages fail to clear bacteria, leading to granuloma formation. The infant's normal immunological parameters exclude primary immunodeficiency, suggesting that immature neonatal immune defenses (e.g., limited macrophage phagocytic activity) may have contributed to XGP development. Its exact pathogenesis remains unclear, but it is widely associated with long-term urinary tract obstruction and bacterial infection. Following renal parenchymal destruction caused by infection, abnormal lipid metabolism and macrophage phagocytosis of lipids leading to foam cell formation are hallmark pathological features (1). Based on disease extent, XGP is classified into three subtypes. Intrarenal (focal) type: Lesions confined to the renal parenchyma without breaching the renal capsule (15%–20% of cases). Perirenal type: Inflammation extending to perirenal fat or fascia, forming perinephric abscesses or inflammatory masses (60% of cases). Diffuse type: Widespread renal involvement, often accompanied by loss of renal function (20% of cases) (2, 3). In this pediatric case, imaging revealed a focal space-occupying lesion in the mid-pole of the left kidney without perirenal infiltration on contrast-enhanced MRI, consistent with intrarenal XGP. This subtype is exceptionally rare in infants, potentially due to early diagnosis and antimicrobial therapy limiting inflammatory spread. Notably, pathological features vary among subtypes: intrarenal XGP often exhibits fibrotic encapsulation of purulent cavities, while diffuse XGP demonstrates extensive necrosis and foam cell infiltration. Although typical cholesterol clefts were absent in the biopsy, abundant neutrophil infiltration and focal necrosis suggested coexisting acute inflammation and chronic granulomatous reactions, aligning with XGP's dynamic pathological process.

Infants often present with systemic symptoms (e.g., fever, poor feeding) rather than classic urinary tract signs (e.g., flank pain, dysuria). This case manifested solely as fever, mimicking sepsis or systemic infections. Ultrasound typically shows mixed-echoic masses, while CT/MRI may reveal rim enhancement or the “bear paw sign”. However, in infants with smaller kidneys and atypical anatomy, differentiation from Wilms tumor or neuroblastoma is challenging (4). The “hyperechoic rim encircling a hypoechoic core” observed here—common in adult XGP—is rarely reported in infants, possibly representing early fibrotic encapsulation. Limited tissue sampling in infants may miss characteristic foam cells. In this case, CD68/CD163 positivity on immunohistochemistry confirmed histiocytic infiltration, supplemented by metagenomic sequencing (Escherichia coli detection). Approximately 85% of XGP cases are linked to E. coli, followed by Proteus and Klebsiella (5). Metagenomic sequencing here identified E. coli as the dominant pathogen (1,044 sequences, 85.78% relative abundance). Potential routes of infection include: Ascending infection (e.g., vesicoureteral reflux or congenital anomalies), though no anatomical defects were found.

Hematogenous spread (neonates' immature barriers permit bacterial seeding).

Lymphatic translocation (theoretically possible but unproven).

Despite negative TB testing, coexisting renal tuberculosis should be considered in endemic regions. EBV (EBER-negative) and fungal (PAS-negative) etiologies were excluded.

Although rare cases link XGP to genetic syndromes (e.g., brachydactyly-mental retardation syndrome), the infant's normal growth and development, absence of dysmorphic features, and negative family history ruled out hereditary etiologies.

Traditionally, nephrectomy was standard for XGP, but image-guided drainage and targeted antibiotics now allow conservative management for localized disease (2). Key factors in this case's success included: Precision antimicrobial therapy: Cefoperazone-sulbactam (renal tissue concentration 60%–80% of serum levels) covers ESBL-producing E. coli. Transition to oral cefdinir (16%–21% bioavailability) ensured urinary tract targeting. While adults require 4–6 weeks of therapy, the infant's rapid metabolism and tissue repair enabled resolution within 4 weeks. Ultrasound-guided biopsy provided diagnostic tissue and potential abscess decompression. However, conservative management carries a 10%–15% relapse risk due to bacterial biofilms or residual pathogens (5). Long-term monitoring of renal function and urine parameters is critical. Laparoscopic drainage may be needed if antibiotics fail or perinephric abscesses develop.

Conventional urine cultures detect pathogens in only 30%–40% of XGP cases due to prior antibiotics or fastidious organisms (5). Renal tissue metagenomics here directly identified E. coli, offering advantages. Simultaneously screens for bacteria, viruses, fungi, and parasites. Identifies pathogens and resistance genes (no ESBL genes detected here, but empirical coverage was chosen). Results within 24 h vs. 3–5 days for cultures.

Limitations include cost, technical demands, and potential detection of colonizers rather than pathogens. Clinical correlation remains vital.

The XGP-related morbidity for a pediatric cohort was 23%, abscess formation (18%) and renal failure, one at the time of nephrectomy and one later on, due to amyloid in the remaining kidney (3%). The XGP-related mortality was non-existent. Although the pathology has excellent prognosis after surgery, an increased risk of UTI, hypertension and, very rarely, renal amyloidosis still exists in the long term, which makes follow-up essential (6). For this infant, follow-up includes: renal ultrasound every 3 months until resolution, then annually until age 5. Regular assessment of serum creatinine, urinary β2-microglobulin, and renal scintigraphy. Voiding cystourethrogram (VCUG) to exclude VUR; prophylactic antibiotics if indicated.

XGP should be considered in infants with fever and renal masses, even without urinary symptoms. Multimodal imaging and metagenomics enhance diagnostic accuracy. Focal XGP may be cured with antibiotics, avoiding nephrectomy. Limited biopsy sampling (no foam cells), incomplete metabolic workup, and short follow-up. The pathogenesis of XGP in this 47-day-old infant likely stems from an interplay of immature host defenses, microbial virulence, and localized tissue response. Escherichia coli, the predominant pathogen here, may have ascended via transient vesicoureteral reflux (VUR) or hematogenous spread, exploiting underdeveloped renal corticomedullary barriers. Notably, the absence of overt urinary tract anomalies on imaging does not exclude microscopic VUR or transient functional obstruction, both of which can potentiate stasis and infection. Histologically, the predominance of neutrophils over foam cells suggests an early phase of XGP, where acute inflammation precedes lipid accumulation and granuloma maturation. This temporal dynamic, coupled with rapid antibiotic response, may explain the absence of classic cholesterol clefts on biopsy. This case underscores that even in the absence of structural anomalies, neonatal renal parenchyma remains vulnerable to aggressive granulomatous responses, necessitating early microbiologic and histopathologic interrogation to avert irreversible damage.

The marked AFP elevation (888.27 ng/ml) warranted differentiation. Neonatal AFP typically declines to <100 ng/ml by 2 months but may transiently rise to 500–1,000 ng/ml in preterm infants or inflammatory states. Inflammatory cytokines may stimulate hepatocyte regeneration. Though imaging showed no tumors, prolonged AFP monitoring is essential (7). This highlights that AFP elevation in infants with infections should not reflexively indicate malignancy.

Pediatric XGP demands heightened suspicion and multidisciplinary collaboration. Integration of imaging, histopathology, and molecular diagnostics enables precision management. Conservative therapy with tailored antibiotics can spare renal tissue in focal cases. Future efforts should establish consensus guidelines to optimize treatment and follow-up protocols.

## Data Availability

The datasets presented in this study can be found in online repositories. The names of the repository/repositories and accession number(s) can be found in the article/Supplementary Material.
